# A digital peer-to-peer learning platform for clinical skills development

**Published:** 2017-02-24

**Authors:** Jesse Basnak, Jennifer Ortynski, Meghan Chow, Emeka Nzekwu

**Affiliations:** 1 University of Calgary, Alberta, Canada; 2 University of Alberta, Alberta, Canada

## Abstract

**Background:**

Due to constraints in time and resources, medical curricula may not provide adequate opportunities for pre-clerkship students to practice clinical skills. To address this, medical students at the University of Alberta developed a digital peer-to-peer learning initiative. The initiative assessed if students can learn clinical skills from their peers in co-curricular practice objective structured clinical exams (OSCEs).

**Methods:**

A total of 144 first-year medical students participated. Students wrote case scenarios that were reviewed by physicians. Students enacted the cases in practice OSCEs, acting as the patient, physician, and evaluator. Verbal and electronic evaluations were completed. A digital platform was used to automate the process. Surveys were disseminated to assess student perceptions of their experience.

**Results:**

Seventy-five percent of participants said they needed opportunities to practice patient histories and physical exams in addition to those provided in the medical school curriculum. All participants agreed that the co-curricular practice OSCEs met this need. The majority of participants also agreed that the digital platform was efficient and easy to use.

**Conclusion:**

Students found the practice OSCEs and digital platform effective for learning clinical skills. Thus, peer-to-peer learning and computer automation can be useful adjuncts to traditional medical curricula.

## Introduction

Pre-clerkship learning of the patient history and physical examination is an integral part of medical education. However, both time and resources are common constraints to providing sufficient learning opportunities for clinical skills within the medical curriculum.^1^,^2^

Peer-to-peer learning can meet this demand.^3^–^6^ Students readily learn from one another and from the preparation of educational material.^7^,^8^ It has been suggested that peer interaction is necessary for optimal learning, in that students who learn in groups significantly outperform those that process information individually.^9^ Peer-to-peer learning also engages medical students in their lifelong role as physician-teachers.^7^

In recent years, studies have shown the efficacy of students as bedside teachers,^7^,^10^ simulated patients,^11^ OSCE examiners,^12^,^13^ and even OSCE designers.^14^ For example, in a study by Haist et al., first-year medical students randomized to physical examination sessions taught by fourth-year students performed equally well as those taught by faculty in objective measures.^10^

At the University of Alberta, first-year medical students formed a club to practice clinical skills and OSCEs. They organized co-curricular history and physical exam practice sessions for their classmates in an OSCE format. They used a digital platform to share documents and automate the process, especially the feedback. This study evaluated student perceptions on learning opportunities within the medical curriculum and peer led co-curricular practice OSCEs.

## Methods

### Case creation

At the beginning of the school year, students were invited to sign up for the club and participate in practice OSCE sessions, and to contribute by writing cases. Case creation was encouraged but not mandatory. Students were given a standardized template for formatting the case. Cases were chosen based on students’ own interests and experiences, but also alignment with the learning objectives of the curriculum. For example, in the cardiology block, students were expected to learn about heart failure. If no cases on heart failure had been written, the club leaders would contact members and request a volunteer to create such a case.

Once a case was written, the club leaders reviewed it for accuracy of content, appropriate length, difficulty, and format. They then sent it to a staff physician content expert. When the edits were finalized, the case was ready for use in a practice OSCE.

### Session sign-up and sign-in

Practice OSCE stations were designed for first-year medical students. Sessions ran at the end of each block in the curriculum, approximately four to eight weeks apart. There were six practice OSCE sessions in total. Two weeks prior to each session, club members were emailed an online sign-up sheet and could select one of three dates to attend.

Once students signed up, the club leaders divided and distributed the cases for that block. A week before the practice OSCE, participants were emailed their case to read, which they would act out as a simulated patient. At the session, students signed in electronically, and their attendance was tracked through a sign-in form.

### Session procedure

There were two students per room, and two to six stations per OSCE session. At each station, one student acted as the physician and one student acted as the patient and assessor. The “physician” could ask questions, perform a physical exam, and request lab tests and imaging. The “patient” would then supply the answers and lab values, or show images from the case using the laptop or phone they had on hand. Stations were timed to mimic OSCEs used by the medical school for assessment purposes.

During or after the interaction, the student-patient filled out an online assessment form for the student-physician, checking off tasks that were successfully completed. A section (e.g. history of presenting illness) was marked as complete if the peer assessor acknowledged completion of all objectives on the checklist. The student-patient provided verbal feedback after the case, using the assessment form to direct the discussion. The assessment form also had an embedded script that automatically sent the results to the learner’s email upon submission, so they could see what was marked as complete or incomplete in each section.

After completing the station and discussing feedback, participants switched roles. The student playing the physician would now play the patient, reading from a different case and filling out a new relevant assessment form. They then rotated through the other stations together until completion of the session in approximately one to two hours.

**Figure 1:**
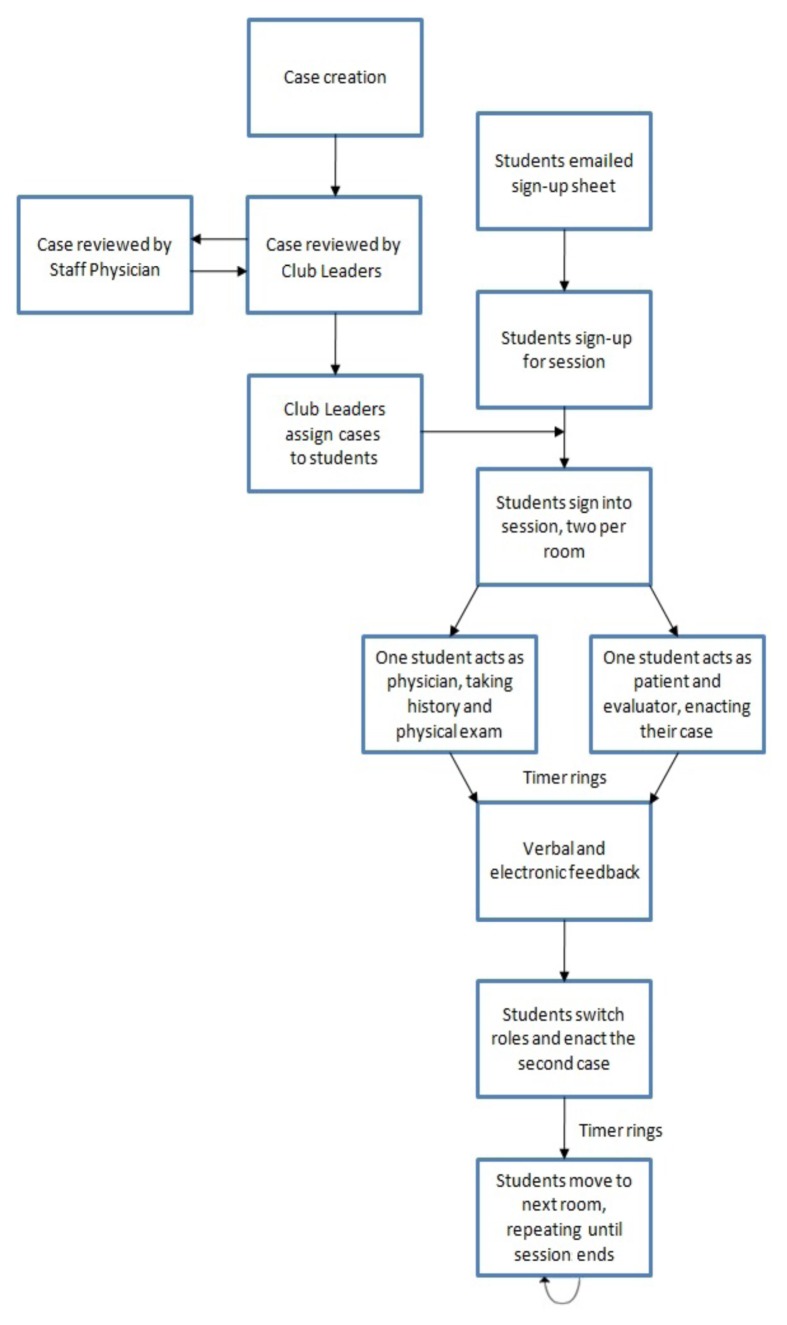
Practice OSCE flow chart

Figure 1 outlines the procedure from case creation to the end of a session. Further details on the procedure and digital platform are available from the corresponding author.

### Surveys

Surveys were developed by the club leaders and distributed to club members via email. Data collection was approved by the University of Alberta’s Research Ethics Board.

To assess student perceptions of the practice OSCE procedure, a session survey (Appendix A) was administered to first-year medical students in the middle of the school year. This time point was chosen to incorporate any changes in the program that participant feedback may have directed. The session survey asked students if they agreed or disagreed with statements on the timing of the sessions, the efficiency of the sign-up and sign-in processes, the ease of following the procedure, and their comfort with acting as a patient and giving peer feedback.

At the end of the year, a second satisfaction survey (Appendix B) determined how first-year medical students perceived learning clinical skills within the medical curriculum, versus from their peers in peer led co-curricular practice OSCEs. Students were asked if they felt they needed more time to practice their histories and physical exams within the curriculum. The survey also asked students whether the practice OSCE sessions met their need to practice histories and physical exams, and were effective for their learning overall.

## Results

### Participation

Of the 165 first-year medical students, 144 (87% of the class) participated in the clinical skills club during the 2012–2013 academic year. Student participants wrote 41 clinical cases throughout the year. Participation in sessions ranged from 22–82%. The most attended session was the endpoint cumulative practice OSCE (118 participants, 82%), which occurred just prior to a mandatory summative OSCE. The least attended sessions were the infectious diseases practice OSCE in the second block and the cardiology practice OSCE in the last block (32 participants each, 22%). Except for the midpoint endocrinology practice OSCE (89 participants, 62%), there was a pattern of lower attendance in the middle of the school year: 94 (65%), 32 (22%), 89 (62%), 32 (22%), 56 (39%), and 118 (82%) in chronologic order.

### Session survey

The midpoint session survey assessed students’ views on the practice OSCE procedure. Forty-nine participants responded (34%). All responders agreed that the timing of the sessions, usually held in the evenings after classes, fit into their schedules. All students also agreed that the online sign-up and sign-in processes were efficient. Forty-three students (88%) believed that the session procedure was easy to follow. Forty-one (84%) felt comfortable acting as a simulated patient in front of their classmates, and 44 (90%) were comfortable giving and receiving feedback from their peers.

### Satisfaction survey

The satisfaction survey at the end of the year evaluated students’ perspectives on learning clinical skills within the medical curriculum, compared to cocurricular practice OSCEs. The response rate was 31% (n = 44). Thirty-three students (75%) felt there were insufficient opportunities to practice patient histories and physical exams in their medical school curriculum. By the end of the school year, all responders felt that the practice OSCEs provided sufficient opportunities to fill this gap in the curriculum. All participants agreed that the sessions were effective for learning clinical skills.

## Discussion

The clinical skills club was established to develop the history taking and physical examination skills of first-year medical students. The practice OSCE sessions had high rates of participation at the beginning and end of the school year, but attendance was lowest midway. This could have been due to students’ increased curricular workload, or confidence in their clinical skills with time, or both. The practice OSCE held just prior to the mandatory OSCE at the end of the year had the highest attendance. This may be an optimal time to run practice OSCEs, as students are motivated to practice with each other before they have to perform in a summative OSCE.

Students also participated in case creation. In the satisfaction survey, one student commented that “it was extremely useful to write up cases.” Writing cases can be a learning exercise in itself, preparing students to be teachers. In a study by Moseley et al. in 2002, senior medical students wrote OSCE cases for their junior peers.^8^ The senior students were surveyed in residency and agreed that the experience better prepared them to teach as residents.^8^

Students in the clinical skills club agreed that the sign-up and sign-in processes were efficient, and the procedure was easy to follow. They could sign in and fill rooms on a “first come, first served” basis, without waiting for other classmates to arrive. Programmed scripts ensured students assigned to the same case did not sign into the same room, minimizing any reorganization by the club leaders. These computer automations likely contributed to the efficiency of the sessions.

Students said they were comfortable acting as the simulated patient in the sessions. This is important, as discomfort could detract from the quality of learning. However, sensitive physical examinations were not included in the practice OSCE cases. This is an obvious limitation of peers acting as patients, and would be a barrier to practicing physical exams in certain blocks, such as urology and gynecology. Students commented that reading from their computer sometimes disrupted the flow of the mock interview. On the other hand, it allowed for multimedia to be instantly available to show the acting physician, should they ask for it.

Students were also comfortable giving and receiving feedback from their classmates. Other studies have demonstrated that most students feel comfortable exchanging peer feedback, and they find such feedback useful.^15^,^16^ Feedback from peers is reportedly more helpful and less intimidating than feedback from instructors.^17^ Fellow students have also been reported to asses one another objectively,^18^ and peer assessment scores have been found to correlate with instructor scores in OSCE settings.^13^,^19^,^20^

Our assessment process provided an online marking checklist for the student-patient to read to the student-physician after they completed the case. In a previous study on medical student competencies, Ferenchick and Solomon used an online assessment and feedback tool with embedded checklists. They found it had high satisfaction, inter-rater reliability, and validity.^21^

Our assessment process was unique, as it used a script to automatically send feedback to the student’s email. Upon submission of the assessment form, a copy was instantly emailed to the student, who could later review what sections of their history and physical exam were incomplete. Kapler et al. have suggested that students benefit from repetitive, delayed feedback, as it forces them to return to previously learned material.^22^ This is a form of spaced re-learning, an established method of memory retention and performance enhancement.^23^,^24^ However, prompt feedback is also necessary, so that students learn from their mistakes and do not consolidate errors into memory.^22^

Previous studies have used medical students as patients and examiners in OSCEs, but these studies did not assess student perceptions on available learning opportunities in the medical curriculum.^11^–^13^,^19^ In our study, students felt there were insufficient opportunities to practice their clinical skills within the University of Alberta curriculum. The perceived insufficiencies are likely due, at least in part, to limitations in time and resources, including standardized patients, as reported at other institutions.^1^,^2^ There are also other curricular priorities to consider besides clinical skills, such as patient-centered care and basic and clinical sciences.

The co-curricular practice OSCEs met our students’ need in learning clinical skills. All students agreed that the sessions gave them enough opportunities to practice their histories and physical exams, and that the sessions were effective for learning clinical skills overall. Thus, our results demonstrate that practice OSCEs and peer-to-peer learning can provide students with opportunities to effectively learn clinical skills alongside the medical curriculum.

However, our study had numerous limitations. Survey answers are subjective and dependent on response rates. The peer evaluation forms were standardized, but they were not reviewed by physicians, and students’ marks were not cross-checked by physicians. Thus, there may be inconsistencies in how students marked each other, and in the accuracy of their feedback. Although students agreed that the sessions were efficient, no objective measures of efficiency, such as time- and cost-effectiveness, were included in the study.

Future research could include measures of efficiency. This may determine if it is worthwhile for medical curricula to incorporate some of the methods used in this initiative, such as automatic electronic feedback. As of the 2016–2017 school year, the clinical skills club is still running at the University of Alberta. The club continues to be well received by pre-clerkship students, although with the usual pattern of falling attendance rates as the year goes on. It would be beneficial to survey medical students on why attendance falls midway through the school year, to better plan practice OSCEs in the future. Further questioning could also focus on the perceived learning benefits of case creation.

Medical students at our institution needed more opportunities to practice their history taking and physical examinations within the medical curriculum. Co-curricular practice OSCEs met this need. Using computer automation to organize and run practice OSCEs may enhance the process. Further studies are needed to validate the utility of this digital platform. Ultimately, our findings further strengthen a growing body of evidence that student-run OSCEs and peer-to-peer learning are effective adjuncts to contemporary medical curricula.

## Appendix A Session Survey

The purpose of this survey is to evaluate the practice OSCE procedure. Please select “agree” or “disagree” for each statement.

### Session time

The timing of the practice OSCE sessions worked well with my schedule.

### Sign-in and sign-up procedure

I found the sign-in and sign-up procedure streamlined and efficient.

### Session procedure

I found the practice OSCE procedure easy to follow.

### Case acting

I felt comfortable acting out the case scenario with my peers.

### Feedback

I felt comfortable giving and receiving peer feedback.

### Additional comments

If you disagreed with any of the above statements, please comment.

## Appendix B Satisfaction Survey

The purpose of this survey is to evaluate clinical skills learning opportunities in the medical school curriculum and in the co-curricular practice OSCEs. Please select “agree” or “disagree” for each statement.

### Curricular learning opportunities

I felt that I needed more time to practice histories and physical exams in the medical school curriculum.

### Practice OSCE learning opportunities

I felt that the practice OSCE sessions provided me with enough opportunities to practice histories and physical exams this school year.

### Overall effectiveness

I felt that the practice OSCE sessions were effective for learning clinical skills overall.

### Additional comments

If you disagreed with any of the above statements, please comment.
